# Assembly and comparative analysis of the complete mitochondrial genome of *Indocalamus longiauritus*

**DOI:** 10.3389/fpls.2025.1599464

**Published:** 2025-06-13

**Authors:** Shinan Liu, Yinuo Zhang, Lijie Li, Dayong Huang, Yonghua Qin

**Affiliations:** ^1^ Guangxi Colleges and Universities Key Laboratory for Cultivation and Utilization of Subtropical Forest Plantation, College of Forestry, Guangxi University, Nanning, China; ^2^ Guangxi Key Laboratory of Superior Timber Trees Resource Cultivation, Guangxi Forestry Research Institute, Nanning, China; ^3^ Guangxi Forestry Inventory & Planning Institute, Protected Areas and Wetland Planning & Research Center, Nanning, China

**Keywords:** *Indocalamus longiauritus*, mitochondrial genome, repeat sequence, predicted RNA editing sites, phylogenetic analysis

## Abstract

*Indocalamus longiauritus*, as a dwarf bamboo holds the ecological and economic significance. Although earlier studies have successfully elucidated its chloroplast (cp) genome, the complete mitochondrial (mt) genome still is uncovered. This study undertook the sequencing, assembly, and comprehensive analysis of the complete mt genome of *I. longiauritus*. Based on the findings, the mt genome contained one circular and two linear contigs with the total length of 491,541bp. Totally, 59 genes were identified, which included 37 protein-coding genes (PCGs), 3 rRNA genes and 19 tRNA genes. In addition, 119 SSRs and 234 dispersed repeats were discovered. We discovered 602 RNA editing sites, with a striking 78.9% of them involving the conversion of hydrophilic amino acid to hydrophobic ones. Furthermore, in the *I. longiauritus* mt genome, 12 genes included 8 PCGs (*petB*, *psbH*, *psbN*, *atpE*, *ndhJ*, *rps4*, *psaB*, and *ndhI*) and 4 tRNA genes (*trnM*-*CAU*, *trnV*-*UAC*, *trnF*-*GAA*, and *trnS*-*GGA*) were found to transfer from the cp genome. Phylogenetic analysis showed a close genetic relationship of *I. longiauritus* with the species *Fargesia qinlingensis* and *I. tessellatus*. Collinearity analysis suggested that significant rearrangements existed in the mt genome of *I. longiauritus*. Selection pressure analysis revealed that more than half of PCGs had Ka/Ks values less than 1. Obviously, certain genes including *rpl2*, *rpl5*, *rpl10*, *rpl14*, *rps2*, *rps11*, *rps12*, *rps14*, *rps19*, and *sdh4* were absent in the mt genomes of *I. longiauritus* and nine relative Poeceae species. Interestingly, the *rpl14* gene was uniquely present in the mt genome of *I. longiauritus*. This study provides a significant genetic resource for the Bambusoideae family, which will facilitate further investigations in the molecular diversity and genetic evolution of bamboos.

## 1 Introduction

Mitochondria are crucial for the synthesis and conversion of energy, which support a variety of cellular processes, making them vital for the growth of plants (Li et al., 2024; Jacoby et al., 2012). These organelles, through the process of phosphorylation, convert biomass energy into chemical energy and participate in essential cellular functions including division, differentiation, and apoptosis (Braun, 2020; Ghifari et al., 2023). Despite being separate from the nucleus, mitochondria possess their own genomes, which are maternally passed down in a haploid (Cheng et al., 2021). These organelles contain their own distinct genomes, which are vital for various biological functions and research purposes (Liu et al., 2025). In accordance with the endosymbiotic theory, it is considered that mitochondria originate from a symbiotic relationship between alpha-proteobacteria and ancestral archaea host cells, evolving into essential organelles within eukaryotic cells (Roger et al., 2017). Furthermore, mitochondrial (mt) genomes and plastidial genomes exhibit maternal inheritance but with significant differences (Zervas et al., 2019). The size of these mt genomes varies considerably among plant species, ranging from 0.25Mb to 11.7Mb (Wu et al., 2022; Sloan et al., 2012; Putintseva et al., 2020). While it has been commonly observed that the mt genomes in plants typically appear as circular structures, more recent studies have revealed their presence in various forms, including multiple branched, linear, circular, branching, or reticular structures (Wu et al., 2015; Hong et al., 2021; Sloan, 2013). The mutation rates in the mt genomes of plants are generally higher than those found in nuclear genomes, primarily due to inadequate the DNA repair mechanisms, causing both duplications and rearrangements (Cole et al., 2018; Kong et al., 2025). In addition to being directly inherited from ancestral mitochondria, plant mitochondrial tRNAs also originate from the migration of sequences from their own chloroplast (cp) genomes (Xiong et al., 2008; Szandar et al., 2022). Therefore, the mt genomes of higher plants harbors a wealth of genetic variation, making them the excellent molecular marker for investigating species origin, evolution, and population genetic diversity (Yang et al., 2022; Liu et al., 2023a).

The Poaceae family, which encompasses around 11,800 species across 12 subfamilies hold significant economic and ecological value (Soreng et al., 2022; Arthan et al., 2025). To date, complete mt genome of over 20 Poaceae species have been sequenced, with genome sizes ranging from 321kb (*Zizania latifolia*) to 704kb (*Tripsacum dactyloides*) (Liu et al., 2023b; Yang et al., 2024; Luo et al., 2024). Moreover, diverse structural forms have been identified within Poaceae, as exemplified by single circular DNA molecules in *Oryza minuta* and *Avena longiglumis* (Asaf et al., 2016; Liu et al., 2023b), and dual circular DNA molecules in *Z. latifolia* and *Setaria italica* (Luo et al., 2024; Zhang et al., 2024a). In contrast, *Fargesia qinlingensis*, a Bambusoideae subfamily species, has a linear mt structure (Wu et al., 2024a). Bamboos, despite being classified as grasses, exhibit tree-like traits, which may be contributed to the different structure in mt genome. However, limited high-quality bamboo mt genomes in public databases hinder the exploration (Wang et al., 2024a). Besides, molecular phylogenetic analyses based on cp genomes and nuclear genes have been widely used to resolve the evolutionary questions in Bambusoideae (Wang et al., 2024a). Studies showed that *Indocalamus* was not monophyletic, which is clustered with *Gelidocalamus*, *Chimonobambusa*, *Bashania*, and *Pseudosasa* (Zeng et al., 2010; Wang et al., 2024a). This underscores the controversy in *Indocalamus* phylogenetic relationships. When compared with the cp genome, the plants’ mt genome is variable in structures (Richardson et al., 2013). Therefore, the analysis of mt genome sequence is crucial for comprehending the evolution of various plant species (Wang et al., 2024b). In this study, we assembled and annotated the mt genomes of *I. longiauritus* from *Indocalamus* genus (Bambusoideae), and further analyze the key genomic features such as codon usage, repetitive sequences, and mt plastid DNAs (MTPTs), RNA editing sites, and the phylogenetic position. Moreover, this will provide a valuable reference for understanding the structural characteristics and evolutionary diversity of the mt genome in the Bambusoideae family.

## 2 Materials and methods

### 2.1 Plant materials sampling, DNA extraction, and sequencing

The young and healthy leaves of *I. longiauritus* were harvested from the bamboo garden at the Guangxi Forestry Research Institute, located in Yongwu district, Nanning, Guangxi Zhuang Autonomous Region (coordinates: 108°20′51"E, 22°55′38"N). Immediately after collection, the samples were flash-frozen in liquid nitrogen and then stored at -80°C for further processing. To extract genomic DNA, the Plant DNAzol Reagent (Invitrogen) was utilized according to the manufacturer’s guidelines. The agarose gel electrophoresis and a NanoDrop spectrophotometer (Thermo Fisher Scientific) were used to evaluate the quality and concentration of the extracted DNA. A 15-kb library was constructed using a SMRTbell Express Template Prep Kit 2.0 (Pacific Biosciences, CA, USA). The construction included DNA shearing, AMPure PB bead purification, ssDNA overhang removal, damage repair, end repair, hairpin adapter ligation, and library bead purification. Following quality control, a SMRTbell library was obtained. The library was sequenced on the PacBio Revio platform (Pacific Biosciences, CA, USA) by Shenzhen Huitong Biotechnology Co., Ltd. The CCS algorithm (version 6.0.0) was employed to process the raw data. To generate highly accurate HiFi reads, the parameters were set as follows: -minPasses 3, -minPredictedAccuracy 0.99, and -maxLength 21,000.

### 2.2 Mitochondrial genome assembly and annotation

The PMAT software (v 1.5.3, -g 3.8G) was used to assemble the PacBio HIFI data that the sequencing data volume and an average fragment length were greater than 7G and 7kb (Bi et al., 2024). Subsequently, the assembly results were visualized and manually refined with Bandage v 0.8.1 software to obtain preliminary results (Wick et al., 2015). Thereafter, the HIFI data were aligned with the assembled sequences using the minimap2 software (Li, 2018). The results were polished through NextPolish v 1.3.1 (https://github.com/Nextomics/NextPolish) and manually adjusted to obtain the final assembly sequences along with the corresponding graph files. The complete mt genome of *I. longiauritus* was later annotated using MITOFY (Alverson et al., 2010) and MFANNOT (Beck and Lang, 2010), and its map was generated with OGDRAW (Greiner et al., 2019).

### 2.3 Relative synonymous codon usage analysis

The PCGs from the mt genome were isolated using Perl scripting. To analyze these sequences, CUSP, an online analytical tool, was used in conjunction with Codon W v1.4.4 software (Sharp and Li, 1986) to calculate the essential nucleotide and codon features, including GC content, relative synonymous codon usage (RSCU), and effective number of codons (ENC). To visualize the data, a scatter plot was constructed using the R package ‘ggplot2’, with ENC values being plotted on the vertical axis and GC3 values on the horizontal axis. The expected ENC curve was derived by the formula: ENC = 2 + GC3 + 29/(GC3² + (1 - GC3)²), as established by Romero et al. (2000). In this plot, data points positioned above or close to the standard curve suggested that codon usage bias is primarily shaped by mutational pressures, whereas points lying below indicated that natural selection exerted a dominant role in shaping codon preferences (Sueoka, 1988). Additionally, a GC plot was generated using the ‘ggplot2’ package, with the average GC content of positions 1 and 2 (referred to as GC12) displayed on the vertical axis and GC3 value on the horizontal axis.

### 2.4 Analysis of repeat sequences

SSRs were identified with the MISA v1.0 tool (Thiel et al., 2003). The motif length of one- to six-nucleotides SSR was set as 10, 5, 4, 3, 3 and 3, respectively. Tandem repeat sequences were detected through TRF v4.09, employing parameters of 2, 7, 7, 80, 10, 50, 2000, -f, -d -m (Benson, 1999). Intersperse repeat sequences were identified with REPuter (Kurtz et al., 2001), with the minimum size and identity thresholds being set at 30bp and 90%, respectively. The results were visualized based on the Circos v0.69-6 (Zhang et al., 2013).

### 2.5 Gene transfer analysis and identification of RNA editing sites

The mt genome of *I. longiauritus* was aligned to its cp genome by blastn v2.9.0, with parameters being set as the -evalue 1E-5 and -word size 7 (Altschul et al., 1990). Next, the results were visualized with the Circos v0.69-6 (Zhang et al., 2013). RNA editing sites in the PCGs were analyzed using the PmtREP program with a cutoff value of 0.2 (Mower, 2005).

### 2.6 Phylogenetic and collinearity analysis

To determine the phylogenetic position of *I. longiauritus*, mt and cp genome sequences were utilized from 17 related species available in NCBI, with *Aconitum kusnezoffii* and *Anemone maxima* being outgroups (**Supplementary Table S1**). Six single-copy orthologous genes were identified and extracted from mt genomes (*ccmFc*, *ccmFn*, *matR*, *mttB*, *rpl16*, and *rps1*) and 48 genes from cp genomes (e.g., *atpA*, *atpB*, *atpE*, *atpF*, *atpH*, *atpI*, *cemA*, *clpP*, and others) using Perl scripts. These sequences were aligned with MAFFT v7.429 (Katoh and Standley, 2013). The resulting alignments were processed in Gblocks 0.91b, applying default parameters with the exception of gap handling. Then, maximum likelihood phylogeny was constructed via IQ-Tree v1.6.12 (Nguyen et al., 2015), employing the GTR+F+R2 model for mt genomes and TVM+F+R2 for cp genomes with a bootstrap of 1000. The comparison of mt genomes across 10 related species, including *I. longiauritus* and *Fargesia qinlingensis*, *I. tessellatus*, *Bambusa oldhamii*, *Paspalum vaginatum*, *Setaria italica*, *Eleusine indica*, *Oryza sativa Indica Group*, *Lolium perenne*, and *Triticum aestivum cultivar Chinese Yumai*, was performed using the Blastn v2.9.0 with the parameters of -evalue 1E-5 and -word size 7 (Altschul et al., 1990). In addition, the concordance factors including gene concordance factors (gCF) and site concordance factors (sCF) were calculated by adopting IQ-TREE v.2.4.0 (Minh et al., 2020). Syntenic relationships were visualized with TBtools v2.119 (Chen et al., 2020).

### 2.7 Ka/Ks and PI analysis

To evaluate the substitution rates of nonsynonymous (Ka) and synonymous (Ks), pairs of homologous genes between *I. longiauritus* and *F. qinlingensis*, *I. tessellatus*, *B. oldhamii*, *P. vaginatum*, *S. italica*, *E. indica*, *O. sativa Indica Group*, *L. perenne*, as well as *T. aestivum cultivar Chinese Yumai* were analyzed. Using ParaAR2.0 with its default settings (Zhang et al., 2012), the homologous sequences were aligned across above species. Subsequently, the Ka and Ks values for each gene were identified through the KaKs_Calculator v2.0 tool with the YN method (Zhang et al., 2006). To conduct a comprehensive comparison of nucleotide variability across the cp genome, MAFFT v7.429 (Katoh and Standley, 2013) was employed to analyze the homologous gene sequences. Finally, DnaSP v 6.0 software was used for calculating the nucleotide diversity (Pi) across the cp genome through a sliding window approach with a step size of 100 and a window length of 200 base pairs (Rozas et al., 2017).

## 3 Results

### 3.1 Features of the *I. longiauritus* mt genome

**Figure 1:**
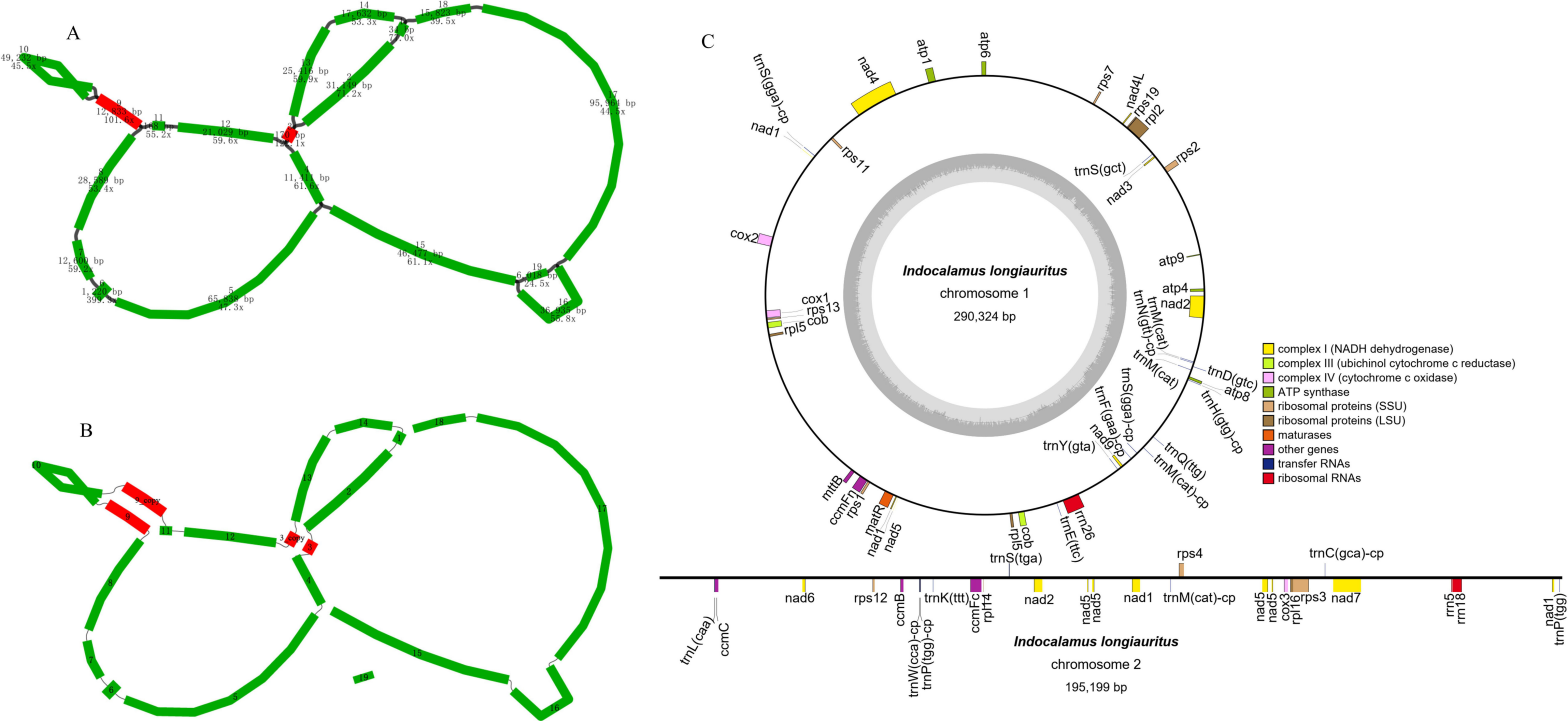
The structural features of *I longiauritus* **(A)** Branched conformation of *I longiauritus* mt genome. **(B)** Three contiguous sequences of *I longiauritus* mt genome after de-catenation. **(C)** The mt genome maps of the *I longiauritus* contig1 and contig2.

The complete mt genome of *I. longiauritus* exhibited a multi-branched conformation (**Figure 1A**) that was 491,541 bp with a GC content of 42.4%. After de-catenation, it was composed of three contiguous sequences (**Figure 1B**). Chromosome 1, chromosome 2, and chromosome 3 had length 290,324 bp, 195,199 bp, and 6,018 bp with GC contents of 44.03%, 44.29%, and 38.33%, separately. Their average depths were 87.11X, 61.27X, and 40.36X for long read, respectively. For subsequent analysis, we restructured Chromosome 1 into a circular molecule following the sequence of contigs 1-2-3-4-5-6-7-8-9-10-9-11-12-3-13-14. Chromosome 2 was also arranged into a linear molecule following the sequence of contigs 15-16-17-18. Chromosome 3 consisted solely of linear fragment, namely contig 19 (**Figure 1B**).

The mt genome of *I*. *longiauritus* included 24 core PCGs, 13 variable PCGs, 3 rRNA genes, and 19 tRNA genes (**Figure 1C**). These PCGs consisted of 9 NADH dehydrogenase genes (*nad1*, *nad2*, *nad3*, *nad4*, *nad4L*, *nad5*, *nad6*, *nad7* and *nad9*), 5 ATP synthase genes (*atp1*, *atp4*, *atp6*, *atp8* and *atp9*), 4 cytochrome c biogenesis genes (*ccmB*, *ccmC*, *ccmFc* and *ccmFn*), 3 cytochrome c oxidase genes (*cox1*, *cox2* and *cox3*), as well as 1 cytochrome c reductase gene (*cob*), 1 maturation enzyme gene (*matR*), and 1 transport membrane protein gene (*mttB*) (**Table 1**). Moreover, the *cob*, *rpl5*, *trnM-CAT* and *trnM-CAT* genes each had two copies (**Table 1**). Furthermore, there were one to four introns for presence in some genes. For instance, the *ccmFc*, *cox2* and *rps3* genes each contained one intron, while *rpl2* contained two; obviously, only *nad7* had four introns, and the remaining genes (*nad1*, *nad2*, *nad4*, and *nad5*) each possessed three introns (**Table 1**).

**Table 1:** Gene composition of the *I. longiauritus* mt genome.

Group of genes		Name of genes
Core genes	ATP synthase	*atp1*, *atp4*, *atp6*, *atp8*, *atp9*
	Cytohrome c biogenesis	*ccmB*, *ccmC*, ccmFc*, *ccmFn*
	Ubichinol cytochrome c reductase	*cob* (2)
	Cytochrome c oxidase	*cox1*, cox2*, *cox3*
	Maturases	*matR*
	Transport membrance protein	*mttB*
	NADH dehydrogenase	nad1****, nad2****, *nad3*, nad4***, *nad4L*, nad5****, *nad6*, nad7****, *nad9*
Variable genes	Ribosomal proteins (LSU)	*rpl14*, *rpl16*, rpl2**, *rpl5* (2)
	Ribosomal proteins (SSU)	*rps1*, *rps11*, *rps12*, *rps13*, *rps19*, *rps2*, rps3* *rps4*, *rps7*
rRNA	Ribosomal RNAs	*rrn26*, *rrn5*, *rrn18*
tRNA	Transfer RNAs	*trnC*-*GCA*, *trnE*-*TTC*, *trnN*-*GAA*, *trnQ*-*TTG*, *trnF*-*GAA*, *trnK*-*TTT*, *trnM*-*CAT* (2), *trnD*-*GTC*, *trnH*-*GTG*, *trnM*-*CAT* (2), *trnP*-*TGG*, *trnW*-*CCA*, *trnP*-*TGG*, *trnY*-*GTA*, *trnL*-*CAA*, *trnS*-*GGA*, *trnS*-*TGA*, *trnS*-*GCT*, *trnS*-*GGA*

The number of asterisks (*) represents intron number. Gene (2): Number of copies of multi-copy genes.

### 3.2 Codon usage analysis

**Figure 2:**
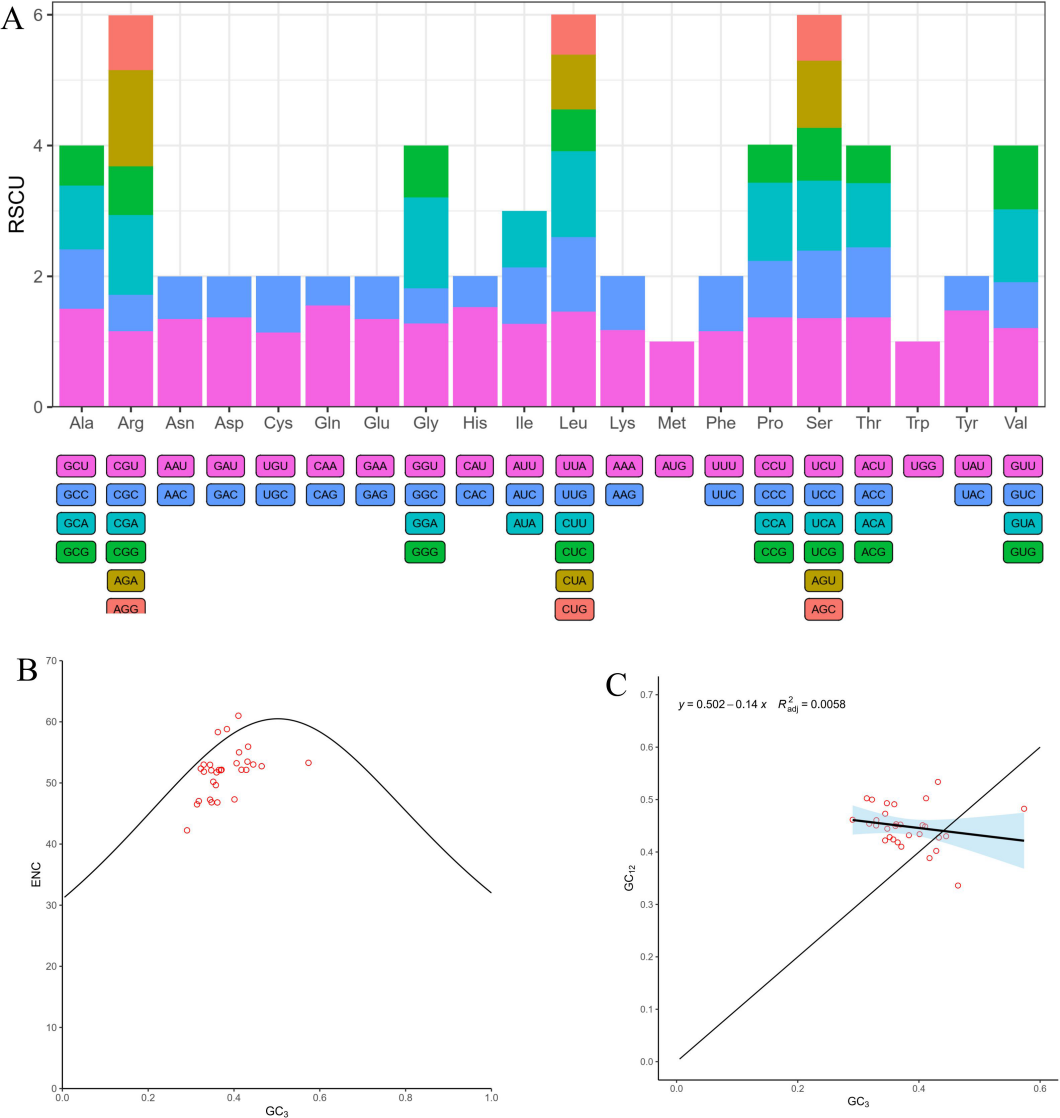
The landscape of codon usage bias in the *I longiauritus* mt genome. **(A)** Relative synonymous codon usage (RSCU) analysis in the *I longiauritus* mt genome. **(B)** The ENC plot of PCGs genes in the *I longiauritus* mt genome. **(C)** The analysis of neutrality plot of PCGs genes in the *I longiauritus* mt genome.

In order to perform the codon usage analysis, the RSCU of PCGs in the mt genome of *I*. *longiauritus* were calculated (**Supplementary Table S2**). The 37 PCGs encoded totally 10,064 codons including termination codons. The most frequently occurring amino acid was leucine (Leu) with 1,049 codons, accounting for 10.42% of the total. The second most frequent one was serine (Ser) with 888 codons, which occupied 8.82%. The termination codon was the least frequent, with 30 occurrences, occupying 0.030% of the total. Moreover, 33 codons exhibited an RSCU value ≥1, among them, 27 codons ended with A or U, while 4 codons (UGG, UUG, UAG, and AUG) ended with G, and 2 codons (UCC and ACC) ended with C (**Figure 2A**, **Supplementary Table S2**). As illustrated in **Supplementary Table S2**, glutamine preferentially utilized the CAA codon, which exhibited the highest RSCU value of 1.55 among these PCGs, followed by histidine (His) and alanine (Ala), showing significant codon usage for CAU (1.53) and GCU (1.5), respectively.

The CUSP analysis indicated that the GC contents at positions GC1, GC2, GC3, and GCall were 29.29% ~ 61.36%, 27.18% ~ 52.91%, 21.57% ~ 54.57%, and 30.46% ~ 56.28%, respectively (**Supplementary Table S3**). Furthermore, their average values were as follows: GC1 (47.69%) > GCall (42.56%) > GC2(42.03%) > GC3 (37.97%) (**Supplementary Table S3**). This indicated that the uneven distribution of GC content at different positions of codons and a preference for A-ended or U-ended in the mt genome of *I*. *longiauritus*. Moreover, the ENC values of different genes ranged from 42.25 to 61, with an average value of 51.78 (**Supplementary Table S3**). Among them, only *rps13* gene had an ENC value below 45, suggesting that these codons in the mt genome had a relatively weak preference in usage. The ENC-plot analysis revealed that 63% of ENC ratios ranged from 0.05 to 0.15, while the remaining proportion (37%) changed from -0.05 to 0.05 (**Figure 2B**). The results indicated that the actual ENC values of most genes deviated significantly from the theoretical ones, implying that the codon usage bias was affected by natural selection rather than mutation. Subsequently, we analyzed the distribution of the gene on a scatter plot illustrating GC3 and GC12 (**Figure 2C**). A low regression line slope (0.14) indicated that the association between GC3 and GC12 was very weak, indicating that mutations exerted a minor role in the formation of codon usage bias in the mt genes of *I*. *longiauritus*.

### 3.3 Repeat sequence analysis

**Figure 3:**
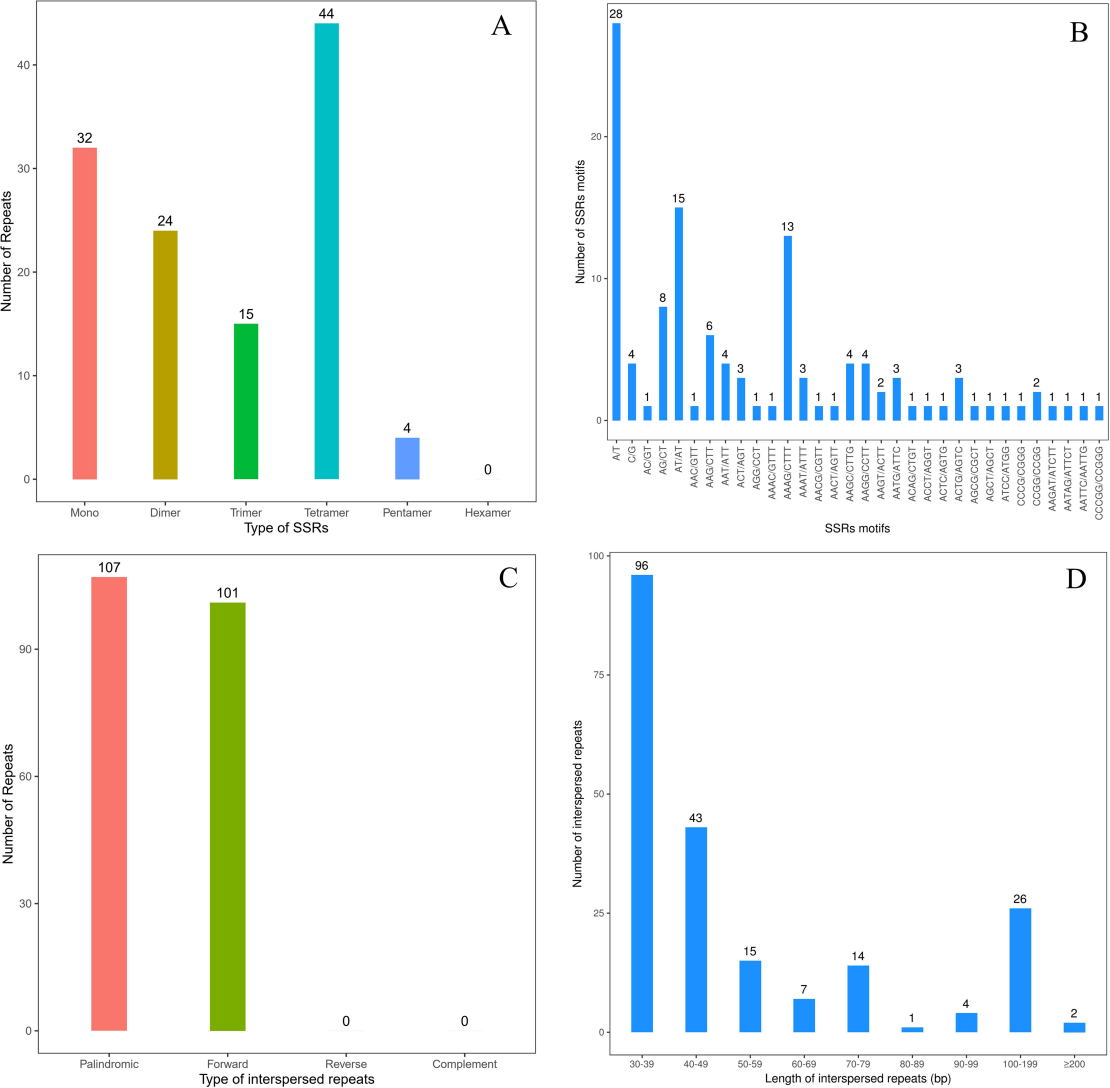
The repeats of *I longiauritus* mt genome. **(A)** Type and number of SSRs repeats. **(B)** Type and number of SSRs motifs. **(C)** Type and number of interspersed repeats. **(D)** The length distribution of interspersed repeats.

Totally 119 SSRs were observed in the mt genome of *I*. *longiauritus*, comprising 32 monomers (26.89%), 24 dimers (20.17%), 15 trimers (12.60%), 44 tetramers (36.97%), 4 pentamers (3.36%), and no hexamers (**Figure 3A**). Next, 28 of the monomeric repeat sequences were classified as repeat type of A or T, identified as the most abundant SSR type, occupying 23.53% of the total (**Figure 3B**). In addition, 15 of the dimeric repeat sequences were AT/AT, recognized as the second most abundant type of SSRs, occupying 12.61% of the total (**Figure 3B**). Moreover, there were 26 tandem repeats in the mt genome with over 75% match and a length of 11 ~ 35bp. In total, 208 pairs of interspersed repeats of at least 30 bp in length were identified, which included 107 pairs of palindromic repeats, 101 pairs of forward repeats, and no pairs of reverse or complement repeats (**Figures 3C, D**).

### 3.4 Prediction of RNA editing sites

**Figure 4:**
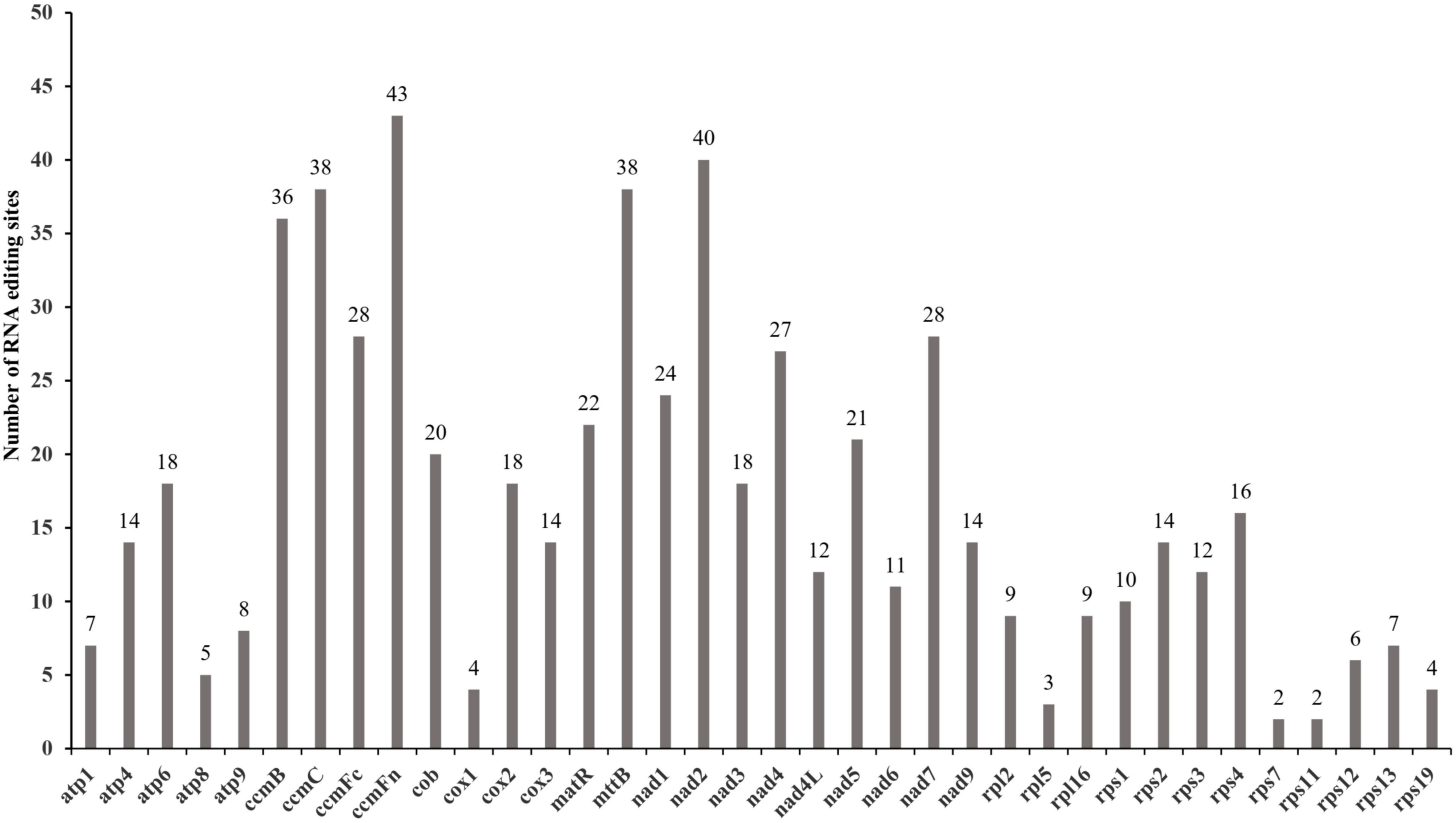
The distribution of RNA editing sites among PCGs in the *I*. *longiauritus* mt genome.

Totally 602 RNA editing sites across 37 PCGs in the *I*. *longiauritus* mt genome were detected. The genes *ccmFn*, *nad2*, *mttB*, *ccmC*, and *ccmB* were identified with 36 to 43 RNA editing sites, each significantly more than the other genes (**Figure 4**). Moreover, the three different types were found in total RNA editing sites (**Table 2**). After RNA editing, 122 positions of amino acids hydrophobicity remained unchanged, while 475 positions were changed from hydrophilic to hydrophobic. Furthermore, only 5 positions of amino acids were changed from hydrophilic to stop. All the identified editing events were the C to T type.

**Table 2:** Prediction of RNA editing sites in the *I. longiauritus* mt genome.

Type	RNA-editing	Number	Percentage (%)
Hydrophilic-hydrophilic	CAC (H) => TAC (Y)	8	20.27
	CAT (H) => TAT (Y)	22	
	CCA (P) => TCA (S)	10	
	CCC (P) => TCC (S)	16	
	CCG (P) => TCG (S)	7	
	CCT (P) => TCT (S)	22	
	CGC (R) => TGC (C)	8	
	CGT (R) => TGT (C)	29	
Hydrophilic-hydrophobic	ACA (T) => ATA (I)	6	78.90
	ACC (T) => ATC (I)	7	
	ACG (T) => ATG (M)	9	
	ACT (T) => ATT (I)	9	
	CCA (P) => CTA (L)	42	
	CCC (P) => CTC (L)	12	
	CCC (P) => TTC (F)	6	
	CCG (P) => CTG (L)	21	
	CCT (P) => CTT (L)	25	
	CCT (P) => TTT (F)	10	
	CGG (R) => TGG (W)	34	
	CTC (L) => TTC (F)	16	
	CTT (L) => TTT (F)	33	
	GCA (A) => GTA (V)	9	
	GCC (A) => GTC (V)	3	
	GCG (A) => GTG (V)	11	
	GCT (A) => GTT (V)	5	
	TCA (S) => TTA (L)	73	
	TCC (S) => TTC (F)	43	
	TCG (S) => TTG (L)	48	
	TCT (S) => TTT (F)	53	
Hydrophilic-stop	CAA (Q) => TAA (X)	2	0.83
	CAG (Q) => TAG (X)	1	
	CGA (R) => TGA (X)	2	
Total		602	100

### 3.5 DNA migration from chloroplast to mitochondria

**Figure 5:**
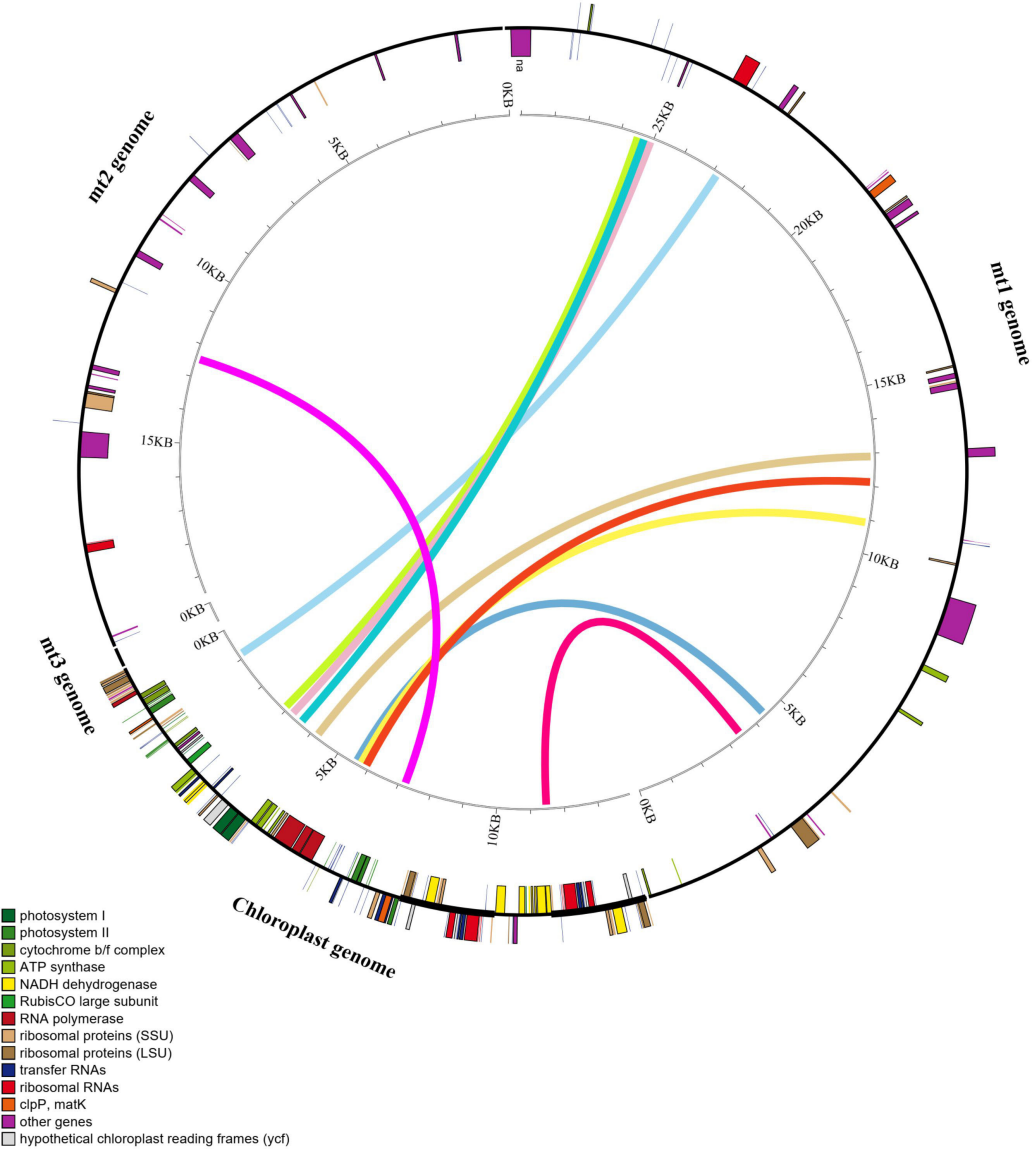
Similar sequences shared between the mt genome and cp genome.

According to sequence similarity analysis, 10 homologous fragments with the more than 1,000 bp in length were screened (**Figure 5**) between the cp and mt genomes of *I*. *longiauritus*. These fragments had a total length of 19,808 bp, while the longest of them was 4,117 bp (**Supplementary Table S4**). Furthermore, the annotated results demonstrated that 12 complete genes were identified in these homologous sequences, transferring from the cp genome to mt genome. They included 8 protein-coding genes (*petB*, *psbH*, *psbN*, *atpE*, *ndhJ*, *rps4*, *psaB*, and *ndhI*) and 4 tRNA genes (*trnM-CAU*, *trnV-UAC*, *trnF-GAA*, and *trnS-GGA*).

### 3.6 Phylogenetic and colinear analysis

**Figure 6:**
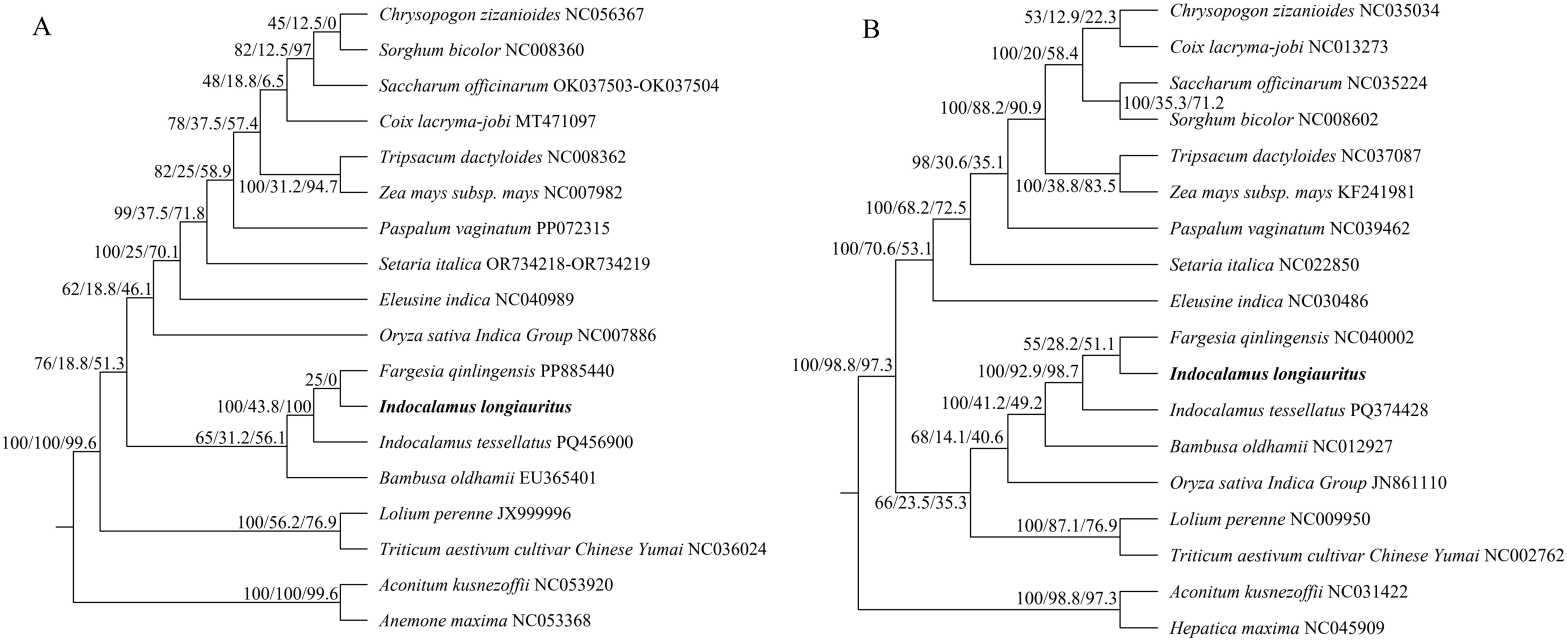
The phylogenetic relationships of *I longiauritus* with other 17 plant species by Maximum likelihood based on the single-copy orthologous genes from mt genomes **(A)** and cp genomes **(B)**. Both trees were annotated with supports which were indicated by concordance factors (UFB/gCF/sCF).

A phylogenetic analysis was perfomed using the six single-copy orthologous genes from 18 species to better understand the evolutionary position of *I*. *longiauritus*. The corresponding information of these species was shown in **Supplementary Table S1**. Among these selected plants, 16 belong to the grass family, including 3 species from Bambusoideae, 9 species from Panicoideae, 2 species from Pooideae, 1 species from Eragrostoideae, and 1 species from Oryzoideae, while 2 species from Ranunculaceae were used as the outgroup. The tree indicated that *I*. *longiauritus* clustered with 3 other species from Bambusoideae, exhibiting a close relationship with *F. qinlingensis* and *I. tessellatus* (**Figure 6A**). In addition, the result was also supported by the phylogenetic analysis based on their cp genomes (**Figure 6B**).

**Figure 7:**
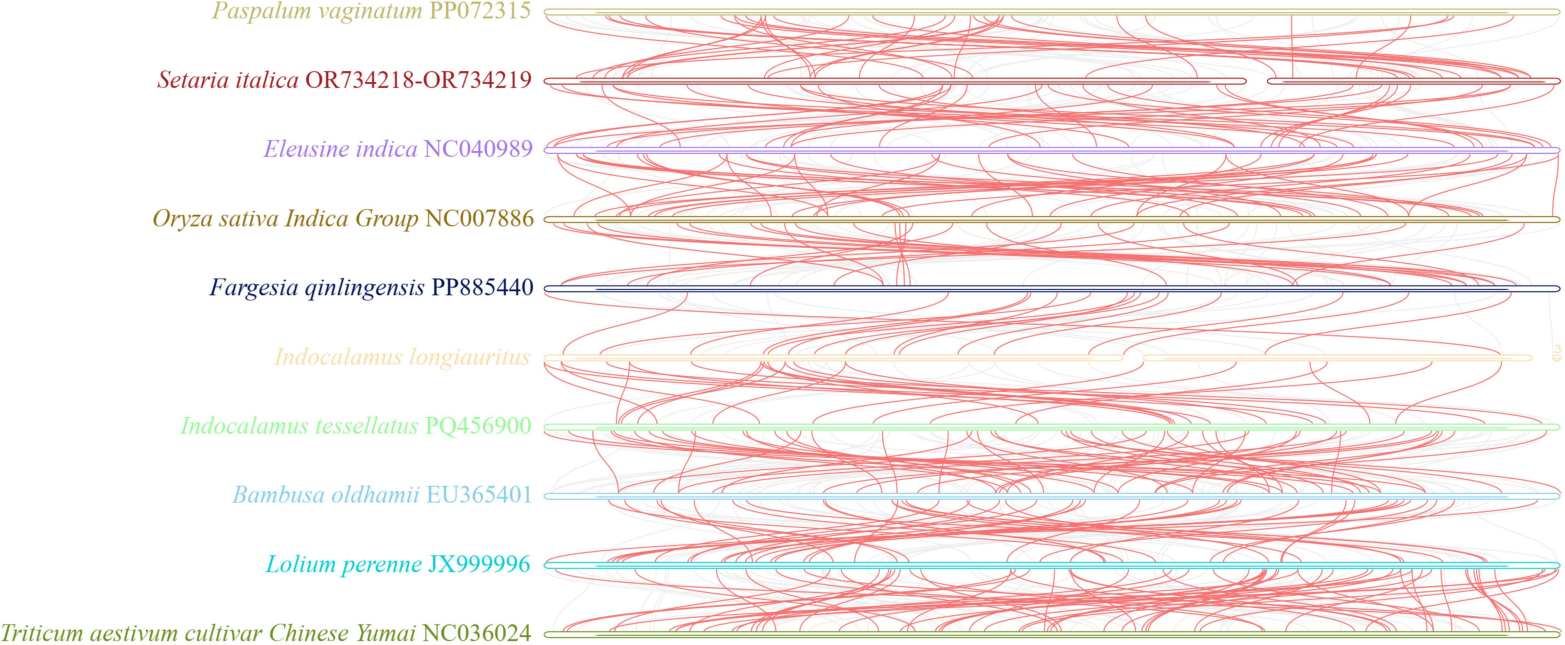
Collinear analysis of the mt genome of *I longiauritus*, *F qinlingensis*, *I tessellatus*, *B oldhamii*, *P. vaginatum*, *S. italica*, *E indica*, *O. sativa Indica Group*, *L. perenne*, and *T. aestivum cultivar Chinese Yumai*. The red arcs represented homologous regions of these mt genomes.

To further assess the mt relationship of *I*. *longiauritus* with its closely related species, 26 homologous genes and their sequence arrangements were compared using the BLAST program (**Figure 7**). The homologous collinear blocks among 9 pairs of species ranged from 30 to 95, with lengths exceeding 1000 bp. The total length of collinear blocks ranged from 371,935 to 590,899 bp from four bamboo species, significantly greater than that of other species. Meanwhile, the order of collinear blocks among different mt genomes was found to be inconsistent. These results indicated that the mt genomes of *I. longiauritus* and its closely related species underwent extensive genome rearrangements, suggesting that the mt genome showed a highly non-conservative structure.

### 3.7 Comparison of the *I. longiauritus* with other nine closely related species (Ka/Ks) and Pi analysis

**Figure 8:**
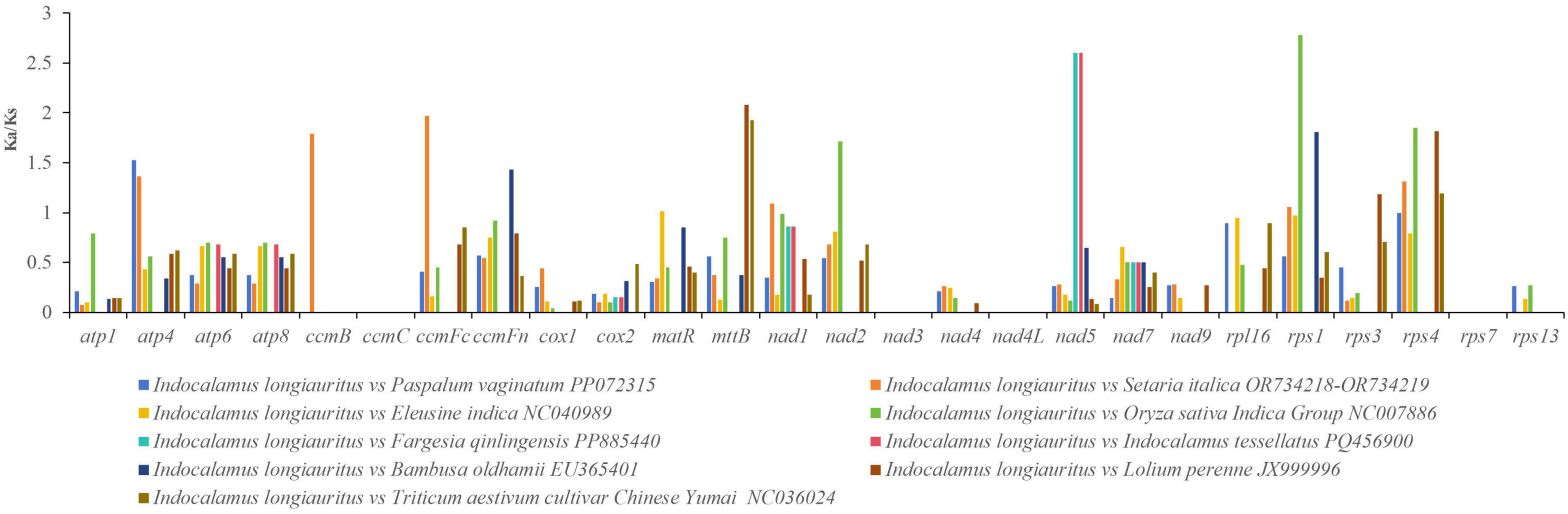
Ka/Ks ratios of 26 PCGs between *I*. *longiauritus* and other nine species.

Based on the result of phylogenetic analysis, nine other Poaceae species were selected for examining of the selective pressure on PCGs. A total of 26 common PCGs which included *atp1*, *atp4*, *atp6*, *atp8*, *ccmB*, *ccmC*, *ccmFc*, *ccmFn*, *cox1*, *cox2*, *matR*, *mttB*, *nad1*, *nad2*, *nad3*, *nad4*, *nad4L*, *nad5*, *nad7*, *nad9*, *rpl16*, *rps1*, *rps3*, *rps4*, *rps7*, and *rps13* were identified and their Ka/Ks values were calculated using *I*. *longiauritus* as a reference (**Figure 8**, **Supplementary Table S5**). The average Ka/Ks value was shown to be less than one. Among these genes, 12 genes including *atp4*, *ccmB*, *ccmFc*, *ccmFn*, *matR*, *mttB*, *nad1*, *nad2*, *nad5*, *rps1*, *rps3*, and *rps4* revealed Ka/Ks values greater than one, while the remaining 11 genes had Ka/Ks values less than one. Moreover, this indicated that the proportions of genes undergoing positive and negative selection were similar.

**Figure 9:**
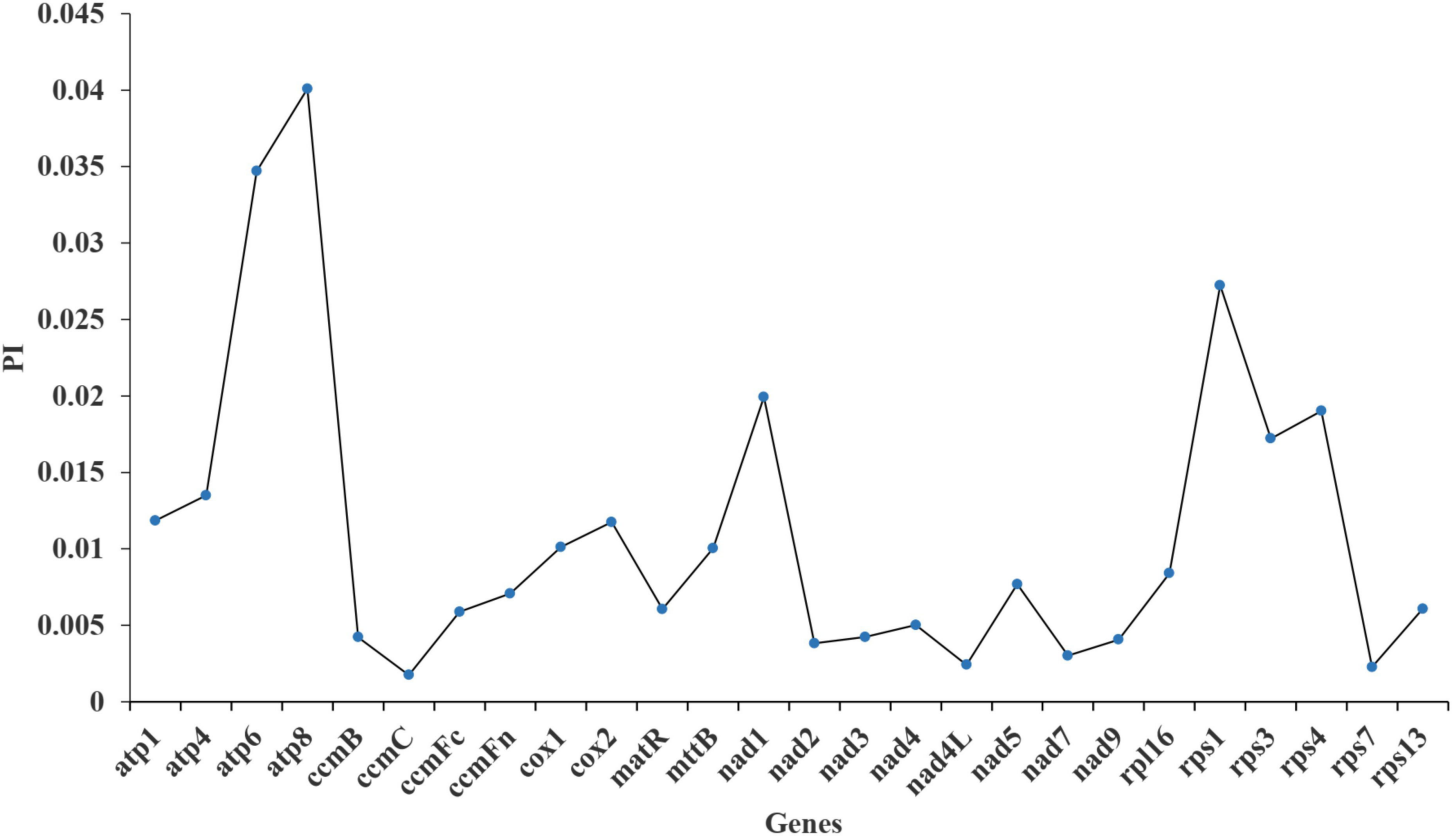
Nucleotide diversity of *I*. *longiauritus* mt genome.

Furthermore, the nucleotide diversity (Pi) of the 26 PCGs within the mt genome was calculated to assess the level of sequence divergence among these 10 species. The Pi values ranged from 0.00175 to 0.04008 (**Figure 9**). The *atp8* gene exhibited the highest Pi value (0.04008), followed by the *atp6* gene (0.0347). In contrast, the *ccmC* and *cox2* genes showed the lowest Pi values (0.00175). All Pi values for the 26 PCGs were all less than 0.05, suggesting a high level of conservation in the nucleotide sequences of these mt genes.

### 3.8 Gene loss and multi-copy gene

**Figure 10:**
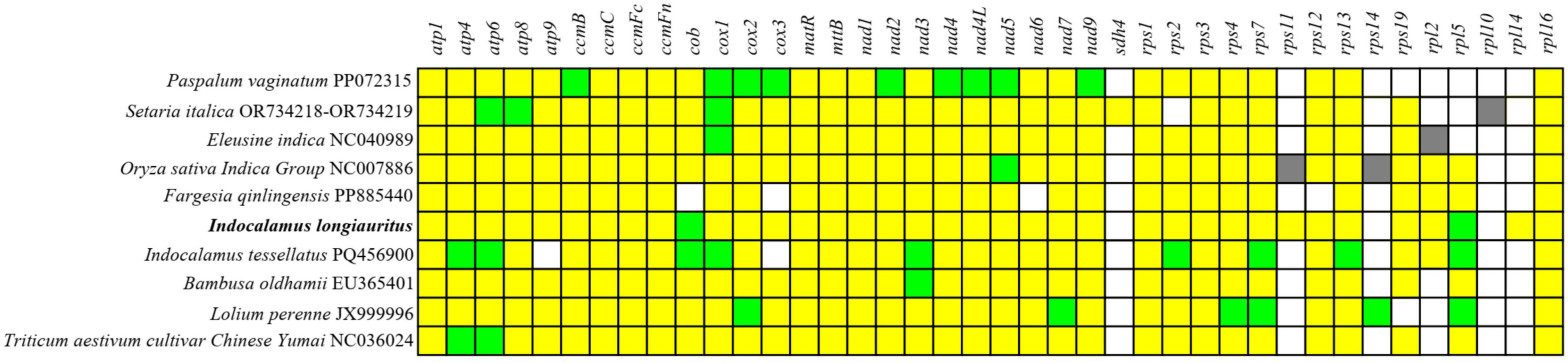
The PCGs distribution of *I*. *longiauritus* and other nine species. Yellow and blue boxes indicated that one and two copies in the mt genomes, respectively. White boxes indicated that the gene was absent in the mt genomes. Grey boxes indicated that the gene was pseudogene.

Based on the phylogenetic tree, other 9 Poaceae plants owned the closer relationships with *I. longiauritus*. So, the distribution of PCGs in their mt genomes was compared. Based on **Figure 10**, most PCGs were conserved, particularly those involved in cytochrome c biogenesis (*ccmB*, *ccmC*, *ccmFc*, and *ccmFn*), maturation enzymes (*matR*), and transmembrane protein genes (*mttB*). On the contrary, the ribosomal protein and succinate dehydrogenase genes exhibited greater variability. Genes including *rpl2*, *rpl5*, *rpl10*, *rpl14*, *rps2*, *rps11*, *rps12*, *rps14*, *rps19*, and *sdh4* were absent in some mt genomes. For example, the *rps10*, *rps14*, and *sdh4* genes were lost in the *I*. *longiauritus* mt genome. Furthermore, the *rpl14* gene and *sdh4* were present only in the mt genome of *I*. *longiauritus* and *S. italica*, respectively. Besides, there were one to nine genes possessing two copies in these mt genomes except for *F. qinlingensis*. Specifically, the *cob* and *rpl5* genes in *I*. *longiauritus* mt genome were demonstrated to contain two copies.

## 4 Discussion

### 4.1 Characterization of the *I. longiauritus* mt genome

In plants, the mt genome is a highly dynamic entity exhibiting significant variation across different species (Petersen et al., 2020). In Poaceae species, relatively few studies have focused on the mt genome structures and their complexities (Evans et al., 2019), so is even Bambusoideae. However, the recent rapid expansion of plant genome sequencing projects has caused a growing number of mt genomes available for comparative analyses. In this study, the sequence, assembly, and analysis of the *I*. *longiauritus* mt genome were carried out. We obtained the sequence of 491,541bp in length with one circular and two linear contigs, which was different from *F. qinlingensis* with three linear contigs (Wu et al., 2024a). Previous studies have demonstrated that the mt genomes of Poaceae species except for Bambusoideae have one or two circular molecules such as *Z. latifolia*, *O. minuta*, and *S. italica* (Zhang et al., 2024a; Luo et al., 2024; Asaf et al., 2016). These results suggest that the mt genome of Bamboo is similar to other species of Poaceae species in sizes, while there is a significant difference in structure. Codon preference can influence amino acid sequences, protein structure and function, influencing an organism’s adaptability and survival (Wu et al., 2024b). It also reflects the evolutionary process and trend of the genome. We identified 33 codons whose RSCU values were greater than 1, indicating that the mt genome displayed a preference in amino acid codon usage, such as CAA in Gln (RSCU=1.55) and CAU in His (RSCU=1.53).

For plants, complete mt genomes were usually consisted of 24 core genes and 17 variable genes. This study compared the distribution of PCGs in the mt genomes of *I*. *longiauritus* and nine relative Poeceae species. The same amount of core genes (24) was also observed in these ten mt genomes, while the number of variable genes varied from 8 to 13. The results demonstrated that most PCGs were highly conserved, especially the genes involved in cytochrome c biosynthesis, cytochrome c reductase, maturase, and membrane transport proteins. By contrast, ribosomal protein genes and succinate dehydrogenase genes exhibited greater variability. Genes including *rpl2*, *rpl5*, *rpl10*, *rpl14*, *rps2*, *rps11*, *rps12*, *rps14*, *rps19*, and *sdh4* are absent in some mt genomes. This is not surprising, as ribosomal protein and succinate dehydrogenase genes are frequently lost or transferred to the nucleus during the evolution of angiosperm mt genomes (Adams and Palmer, 2003; Ueda and Kadowaki, 2012). Noteworthily, four mt genomes of bamboo all lost *sdh4*, *rps14*, and *rpl10*. Besides, only the mt genome of *I*. *longiauritus* had the *rpl14* gene while only that of *I. tessellatus* lost the *atp9* gene. Compared with other three bamboos, only *F. longiauritus* lost *cob*, *nad6*, and *rps12*. These findings showed that the PCGs of mt genomes among four bamboos exhibited diversity, potentially serving as the scientific references for species identification.

The RNA editing phenomenon is one of the essential steps in gene expression for the plant mt genomes, which is widely present in higher plant mitochondria (Small et al., 2023). We acquired 602 RNA editing sites within all the PCGs in the *I*. *longiauritus* mt genome, such figure was extremely greater than that reported in the same subfamily such as *F. qinlingensis* (482) and different subfamilies including *S. italica* (417), *Z. latifolia* (93), and other species (0~35) (Yang et al., 2024; Wu et al., 2024a; Zhang et al., 2024a). All the RNA editing sites were the C-T (U) types, and the finding was similar as the identification results from other plant mt genomes (Gong et al., 2024). Number RNA-editing events in the mitogenome result in the conversion of hydrophilic amino acids to hydrophobic ones (Zhang et al., 2024b). Here, 78.9% of amino acids were changed from hydrophilic to hydrophobic in the mt genome of *I*. *longiauritus*. The increase in hydrophobic amino acids can improve the overall stability of protein structures of the *I*. *longiauritus* mt genome, as hydrophobic interactions exert a vital role in the folding and stability of proteins (Zhang et al., 2024b).

### 4.2 Repeat sequences and nucleotide diversity analysis

Current studies have indicated that numerous different types of repetitive sequences mediate the high-frequency recombination within or between mt DNA molecules in higher plants, making them the primary cause of mt genome structural diversity (Gualberto and Newton, 2017). Here, 109 SSRs were identified in the mt genome of *I*. *longiauritus*. Though the monomeric, dimeric, trimeric, tetrameric, pentameric, hexameric and compound SSRs exist in the Poeceae family, differences are observed among species. For example, *E. inadica* has mono-, di-, tri-, and compound nucleotide repeats (Hall et al., 2020), while *Aegilops* sp*eltoides* possesses mono- to hexa- types (Li et al., 2023). However, SSRs of *I*. *longiauritus* included mono- to penta- types, the same as those of *F. qinlingensis* (Wu et al., 2024a). Additionally, 234 dispersed repeats were detected in our study, and the number was greater than that obtained in *F. qinlingensis* (195), while both species were similar in the distribution of different length (Wu et al., 2024a). These findings may explain why the mt genome of *I. longiauritus* is larger than that of *F. qinlingensis*. The larger Pi values of genes showed the higher variation of nucleotide sequences, potentially serving as the molecular markers for distinguishing different species (Gong et al., 2024). In addition, our results revealed that the Pi value of the *atp8* gene was the largest, suggesting that it evolved more rapidly than other genes. As a component of the oxidative phosphorylation complex on the inner mitochondrial membrane, *atp8* is vital for energy production (Weiss et al., 2012). Due to its essential role, this gene may have been subjected to intense natural selection pressure, leading to a high degree of diversity (Zhao et al., 2022). Bamboo, a plant with wide distribution and strong adaptability, may face to different selection pressures in different ecological environments (Li and Sun, 2024), which can contribute to the increased diversity of the *atp8* gene. The highly variable *atp8* gene can be applied for molecular markers as the mt genome analysis in *I*. *longiauritus*.

### 4.3 Phylogenetic and evolutionary analyses of *I. longiauritus*

The mt genome exhibits a lower evolutionary rate and fewer repetitive events, which can provide a clear homologous gene relationship, effectively addressing the deep nodal compounds in angiosperm phylogeny, and providing an important example for angiosperm phylogeny and evolution studies (Palmer and Herbon, 1988; Drouin et al., 2008; Lin et al., 2022, 2025). In this study, the phylogenetic tree was also constructed on the basis of the mt genomes of 18 species. *I. longiauritus* with three bamboos were clustered into a group, conforming to the reported by Wu et al. (2024a). These results indicated that the mt genome data can be effectively applied in distinguishing bamboo from other species from Poaceae. Noteworthily, that *I. longiauritus* was more closely related to *F. qinlingensis* rather than *I. tessellatus* from the same genus. To further confirm this result, their relationships were supported by the constructed tree according to their cp genomes (**Figure 6**). These findings further corroborated the perspective that *Indocalamus* was generally clustered into several lineages and other genera based on nuclear or cp genome data (Gao et al., 2025). Collinearity analysis demonstrated that the substantial rearrangements existed in the mt genome of *I. longiauritus*, leading to the highly variable structure when compared with its close relatives. Furthermore, this phenomenon exerted a crucial role in the evolution and diversification of the mt genome, as explained by Li et al. (2023). Certain regions of the *I. longiauritus* mt genome display no homology with those of other species, underscoring their distinct presence within this particular mt genome. This remarkable finding has important implications for future studies on the genetics, growth, and development of *I. longiauritus*. These insights may be beneficial for deepening our understanding of the unique biological characteristics of this species and contribute to broader studies in evolutionary genetics. The transfer of DNA sequences between cp and mt genomes is a common phenomenon in plant mt genomes (Straub et al., 2013).

The Ka/Ks ratios are of great significance for reconstructing the phylogeny and understanding the evolutionary dynamics of protein-coding sequences in closely related species (Fay and Wu, 2003). During the evolution in plants, most of mt genes exhibit negative selections with Ka/Ks ratios <1. In this study, 14 of the shared PCGs had Ka/Ks <1, which were obtained by analyzing the Ka/Ks values between *I. longiauritus* and nine related species. This was consistent with other Poeceae species such as *Z. latifollia* (Luo et al., 2024). It was indicated that these PCGs in the mt genome of Poeacea species were highly conserved during evolution. Nevertheless, other genes such as *atp4*, *ccmB*, *ccmFc*, *ccmFn*, *matR*, *mttB*, *nad1*, *nad2*, *nad5*, *rps1*, *rps3*, and *rps4* were more than 1 with Ka/Ks ratio, suggesting positive selection during the evolution. Particularly, the *rps1*, *nad5*, and *mttB* exhibited a higher Ka/Ks value greater than 2, which might play critical roles in evolutionary history and essential life activities, possibly related to environmental stress (Wang et al., 2024b). These will be advantageous for understanding the adaptability of bamboo species, identifying selection factors, evaluating adaptation potential, and investigating heredity and evolution.

## 5 Conclusion

*I. longiauritus* is a bamboo species that holds economic and ecological significance (Bai et al., 2011). In this study, through the integration of multiple sequencing techniques and in-depth bioinformatics analyses, we successfully carried out the assembly and annotation of the mt genome of *I. longiauritus*. When compared with other species in the Poaceae family, the mt genome of *I. longiauritus* exhibits both conservation and specificity in its characteristics. Phylogenetic analyses based on mt and cp genomic datasets have revealed discrepancies regarding the evolutionary position of this species within its taxonomic clades. These results provide foundational insights into genetic traits, molecular variations, taxonomic classification of *I. longiauritus*. Moreover, this research will serve as a reliable genomic resource for future studies on the systematic evolution of the Bambusoideae family.

## Data Availability

The chloroplast genome datasets of *I. longiauritus* with the accession number PV366311 and its mitochondrial genome datasets with accession number PV366312, PV366313, and PV366314 have been deposited in the GenBank of NCBI. The BioProject, BioSample, and SRA numbers of raw sequencing data were PRJNA1265797, SAMN48631790, and SRR33649504, respectively, were also deposited in the GenBank of NCBI.
